# An Iterative Method for Predicting Essential Proteins Based on Multifeature Fusion and Linear Neighborhood Similarity

**DOI:** 10.3389/fnagi.2021.799500

**Published:** 2022-01-24

**Authors:** Xianyou Zhu, Yaocan Zhu, Yihong Tan, Zhiping Chen, Lei Wang

**Affiliations:** ^1^College of Computer Science and Technology, Hengyang Normal University, Hengyang, China; ^2^College of Computer Engineering and Applied Mathematics, Changsha University, Changsha, China

**Keywords:** key protein, entropy, linear neighborhood similarity, iterative method, multi-feature fusion

## Abstract

Growing evidence have demonstrated that many biological processes are inseparable from the participation of key proteins. In this paper, a novel iterative method called linear neighborhood similarity-based protein multifeatures fusion (LNSPF) is proposed to identify potential key proteins based on multifeature fusion. In LNSPF, an original protein-protein interaction (PPI) network will be constructed first based on known protein-protein interaction data downloaded from benchmark databases, based on which, topological features will be further extracted. Next, gene expression data of proteins will be adopted to transfer the original PPI network to a weighted PPI network based on the linear neighborhood similarity. After that, subcellular localization and homologous information of proteins will be integrated to extract functional features for proteins, and based on both functional and topological features obtained above. And then, an iterative method will be designed and carried out to predict potential key proteins. At last, for evaluating the predictive performance of LNSPF, extensive experiments have been done, and compare results between LNPSF and 15 state-of-the-art competitive methods have demonstrated that LNSPF can achieve satisfactory recognition accuracy, which is markedly better than that achieved by each competing method.

## Introduction

In the past few years, with the development of high-throughput and bioinformatics technologies, recognition of potential key proteins based on protein-protein interaction (PPI) networks has become a new research hotspot (Dai et al., 2021; Zhang et al., 2021). Essential proteins play an important role in cell growth and regulation, and researches on essential proteins can deepen the understanding of biological life processes. Existing key protein prediction methods can be roughly divided into two categories: one is based on the topological characteristics of PPI networks and the other is based on the fusion of topological structures of PPI networks and biological information of protein such as the gene expression data, the subcellular localization data, the homologous data, and the gene ontology of protein. For example, based on topological characteristics of PPI networks, Li et al. (2015) proposed a method called LAC, in which, the local average connectivity of nodes in the PPI network was adopted to estimate the essentiality of proteins. Qi and Luo (2016) introduced a model named LID by measuring the importance of proteins by the local interaction density between neighboring nodes in the PPI network. Lin designed two predictive models called MNC (maximum neighborhood connectivity) and DMNC (density of maximum neighborhood connectivity) based on the maximum neighborhood connectivity and density of maximum neighborhood connectivity of modes in the PPI network separately (Lin et al., 2011). In addition, researchers have proposed a series of methods to identify key proteins based on the centrality of nodes in PPI networks, such as DC (degree centrality) (Hahn and Kern, 2005), EC (eigenvector centrality) (Bonacich, 1987), CC (closeness centrality) (Wuchty and Stadler, 2003), IC (information centrality) (Stephenson and Zelen, 1989), SC (subgraph centrality) (Estrada and Rodríguez-Velázquez, 2005), BC (betweenness centrality) (Joy et al., 2005), and NC (neighbor centrality) (Wang et al., 2012). In all these methods, since only topological characteristics of PPI networks were considered, then unknown interactions between proteins might greatly affect the identification accuracy of potential key proteins. Hence, to improve the recognition accuracy, some other methods based on the fusion of biological information and topological features were proposed successively. For instance, Tang and Li proposed two methods called WDC (weighted degree centrality) (Tang et al., 2014) and PEC (integration ECC and Pearson correlation) (Li et al., 2012), respectively, by fusing topological features of PPI networks with gene expression information of proteins to measure the importance of proteins. Peng et al. (2012) designed two methods, namely, UDoNC (united the domain features and the normalized ECC) and ION (integration of the properties of orthologous and the features of neighbors) (Peng et al., 2015a), through combining homology and domain information of proteins with topological features of PPI networks separately. Zhang et al. (2013) introduced a prediction model called CoEWC by integrating topological characteristics of PPI networks with co-expression characteristics of proteins in gene expression profiles. Li et al. (2018) proposed a method named subnetwork partition and prioritization by fusing subcellular localization information of proteins with PPI networks. Zhao et al. (2019) designed an iterative computing method called RWHN by combining homology, domain, and subcellular localization information of proteins with topological features of PPI networks. Zhao et al. (2014) proposed a prediction method called POEM by integrating gene expression data of proteins and topology features of PPI networks. Lei et al. (2020) designed a method based on gene expression data and Drosophila optimization algorithm (FOCA), which combines PPI network, subcellular localization, gene ontology annotation, gene expression data, and artificial fish swarm optimization (AFSO) algorithm (Lei et al., 2016) to predict key proteins. In addition, a prediction method based on the combination of a learning system and specific scoring matrix was proposed by Wang (Wang et al., 2017), and a prediction method based on the deep learning model proposed by Chen (Chen et al., 2019). Chen et al. (2020) proposed an identification method called NPRI by integrating heterogeneous networks. Dai et al. (2020) identified key proteins based on PPI network embedding. Zhang et al. (2019) proposed a method by fusing dynamic PPI networks. Sun et al. (2021) designed an iterative method called IoMCD (iteration based on multiple characteristic differences) based on cross-entropy. Li et al. (2020) proposed an iterative method called CVIM (character vector iteration method) based on the fusion of topological structures of PPI networks and functional characteristics of proteins.

Experimental results show that the fusion of network topological features and biological information of proteins can improve the accuracy of identifying potential key proteins effectively. However, in most existing methods, due to the limited categories of topological structures of PPI networks and functional characteristics of proteins fused, the predictive performances of these methods are not satisfactory. Hence, in this study, through combining a series of topological features of PPI networks and abundant biological information of proteins, a new predictive method called LNSPF (linear neighborhood similarity-based protein multifeatures fusion) is proposed to identify potential key proteins. In LNSPF, an original PPI network will be constructed first based on known PPI data downloaded from benchmark databases, and then, topological features will be extracted from the original PPI network. Next, the protein nodes in the original PPI network are defined as data points, the protein gene expression data are defined as the characteristics of the corresponding data points, and the data points are reconstructed to calculate the linear neighborhood similarity between the data points in the feature space. After that, subcellular location and homologous information of proteins will be integrated to extract functional features for proteins. At last, based on both functional and topological features extracted above, an iterative method will be designed to predict key proteins. Experimental results show that LNSPF can achieve reliable prediction accuracies of 100%, 90%, and 87% in top 1%, 5%, and 10% ranked key proteins separately based on the GAVIN database, which is markedly superior to 15 state-of-the-art competitive methods, namely, DC (Hahn and Kern, 2005), CC (Wuchty and Stadler, 2003), IC (Stephenson and Zelen, 1989), SC (Estrada and Rodríguez-Velázquez, 2005), BC (Joy et al., 2005), NC (Wang et al., 2012), PEC (Li et al., 2012), LAC (Li et al., 2015), COEWC (Zhang et al., 2013), POEM (Zhao et al., 2014), ION (Peng et al., 2015a), TEGS (Li et al., 2018), RWHN (Zhao et al., 2019), IoMCD (Sun et al., 2021), and CVIM (Li et al., 2020) simultaneously.

## Materials and Methods

**FIGURE 1:**
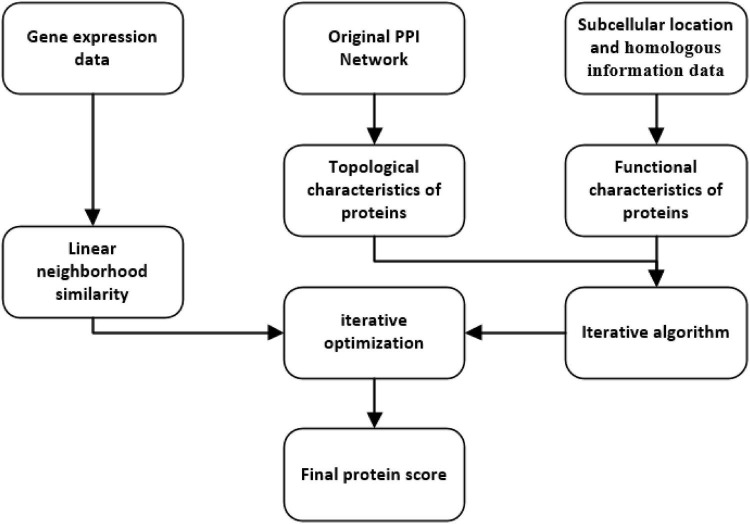
Flowchart of the LNSPF.

As shown in Figure 1, the process of LNSPF consists of the following four main steps:

Step 1: First, based on known PPI data downloaded from the benchmark database, an original PPI network is constructed, from which, topological features, namely, degree, two hops degree, and triangle are extracted successively.
Step 2: Next, subcellular location and homologous information of proteins will be integrated to extract functional features for proteins.
Step 3: Moreover, based on the topological and biological properties obtained above, an iterative method is designed to estimate the importance of proteins.
Step 4: At last, based on the gene expression data downloaded from the benchmark database, the score was further optimized by using linear neighborhood similarity.

### Extraction of Functional Features for Proteins

Let *G* = (*V*, *E*) denote the original PPI network constructed from a dataset of known PPIs downloaded from any given benchmark database *D*, *V* = {*p*_1_, *p*_2_, ⋯ *p*_*N*_} represent a set of different proteins, and *E* = {*e*(*p*_*i*_, *p*_*j*_)|*p*_*i*_, *p*_*j*_ ∈ *V*} represent a collection of edges between proteins in *G*. Here, if and Based a known interaction between any two given proteins in *V*, there is a side *e*(*p*_*i*_, *p*_*j*_) between them. Obviously, based on the original PPI network *G*, we can obtain a *N* × *N* dimensional adjacency matrix *A* = (*a*_*ij*_)_*N*×*N*_, where there is *a*_*ij*_ = 1, if and only if there is an edge *e*(*p*_*i*_, *p*_*j*_) between *p*_*i*_ and *p*_*j*_, otherwise, there is *a*_*ij*_ = 0.

For any given protein *p*_*i*_ in *G*, let *NG* (*p*_*i*_) denote the set of nodes neighboring to *p*_*i*_ in *G*, then it is obvious that there is:


(1) $$NG\left(p_{i}\right)=\left\{p_{j}|\exists e\left(p_{i},p_{j}\right)\in E,p_{j}\in V\right\}$$

According to Equation 1, it is easy to know that the nodes in *NG* (*p*_*i*_) are one-hop from *p*_*i*_ in *G*, for convenience, we define *NG* (*p*_*i*_) as the set of one-hop neighbors of *p*_*i*_ in *G*, based on which, we can obtain a new set of two-hops neighbors of *p*_*i*_ in *G* as follows:


(2) $$THNG\left(p_{i}\right)=\left\{p_{j}|\exists e\left(p_{j},p_{k}\right)\in E,p_{k}\in NG\left(p_{i}\right)\right\}$$

Where |*NG*(*p*_*i*_)| denotes the number of different nodes in the set *NG*(*p*_*i*_).

According to Equations 1, 2, based on the fact that key proteins and their neighbors often form tight junction clusters (Li et al., 2015; Peng et al., 2015a), we can define two kinds of topological properties for any given protein *p*_*i*_ in *G* as follows:


(3) $$TP_{1}\left(p_{i}\right)=\sum_{p_{j}\in NG\left(p_{i}\right)}TZ_{1}\left(p_{i},p_{j}\right)$$

(4) $$TP_{2}\left(p_{i}\right)=\sum_{p_{j}\in NG\left(p_{i}\right)}TZ_{2}\left(p_{i},p_{j}\right)$$

Where,


(5) $$TZ_{1}\left(p_{i},p_{j}\right)=\left\{ \begin{array}{cc} \frac{\left|NG\left(p_{i}\right)\cap NG\left(p_{j}\right)\right|}{\left|NG\left(p_{i}\right)\right|}; & p_{j}\in NG\left(p_{i}\right)\\ 0; & \text{otherwise} \end{array}\right.$$

(6) $$TZ_{2}\left(p_{i},p_{j}\right)=\left\{ \begin{array}{cc} \frac{\left|THNG\left(p_{i}\right)\cap NG\left(p_{j}\right)\right|}{\left|THNG\left(p_{i}\right)\right|}; & p_{j}\in NG\left(p_{i}\right)\\ 0; & \text{otherwise} \end{array}\right.$$

From observing Equations 3, 4, it can be seen that, for any two given proteins *p*_*i*_ and *p*_*j*_ in *G*, the more the number of common one-hop or two-hops neighboring nodes between them, the bigger the values of *TZ*_1_(*p*_*i*_, *p*_*j*_) and *TZ*_2_(*p*_*i*_, *p*_*j*_) will be. Hence, it is obvious that *TZ*_1_(*p*_*i*_, *p*_*j*_) and *TZ*_2_(*p*_*i*_, *p*_*j*_) can to a certain extent reflect the tightness and the aggregation degree between *p*_*i*_ and *p*_*j*_, respectively.

### Extraction of Functional Features for Proteins

Key proteins tend to connect with each other rather than exist independently, and the key of proteins is usually expressed through protein complexes or functional modules, rather than a single protein (Min et al., 2017). Existing studies have shown that key proteins are closely related to the subcellular structures of proteins (Peng et al., 2015b; Li et al., 2016; Fan et al., 2017). In this section, we will adopt the subcellular locations to extract functional features for proteins. First, for any given protein *p*_*i*_, let *Sub*(*p*_*i*_) denote the set of different subcellular locations relating to *p*_*i*_, and |*Sub*(*p*_*i*_)| represent the number of different elements in *Sub*(*p*_*i*_), then, we can calculate one kind of functional property for p_i_ as follows:


(7) $$FP_{1}\left(p_{i}\right)=\frac{\sum_{p_{j}\in NG\left(p_{i}\right)}TZ_{3}\left(p_{i},p_{j}\right)}{\left|NG\left(p_{i}\right)\right|}+1$$

Where,


(8) $$TZ_{3}\left(p_{i},p_{j}\right)=\left\{ \begin{array}{cc} \frac{{\left|Sub\left(p_{i}\right)\cap Sub\left(p_{j}\right)\right|}^{2}}{\left|Sub\left(p_{i}\right)\right|*\left|Sub\left(p_{j}\right)\right|}; & \\ \left|Sub\left(p_{i}\right)\right|*\left|Sub\left(p_{j}\right)\right|>0\; & \\ 0;otherwise\; & \end{array}\right.$$

In addition, in the study of Peng et al. (2012), key proteins were proved to be relatively conserved. Through whether each protein has homology, the homology score of each protein is obtained to indicate the degree of conservation of each protein. Based on the homology information of proteins, for any given protein *p*_*i*_, let *os*(*p*_*i*_) denote the homology fraction of *p*_*i*_, then we can obtain another kind of functional property for p_i_ as follows:


(9) FP2(pi)=os(pi){os(pj)}pj∈Vmax

### Construction of Linear Neighborhood Similarity-Based Protein Multifeatures Fusion

#### Initial Iteration

For generality, supposing that we have extracted *M*_1_ different topological features (such as *TP*_1_, *TP*_2_,…, *TP*_*M*_1__) and *M*_2_ different functional features (such as *FP*_1_, *FP*_2_, …, *FP*_*M*_2__), moreover, there is *M*_1_ + *M*_2_ = *M*, then, for any given protein *p*_*i*_, we can construct a feature vector for it as follows:


(10) $$\begin{array}{c} V_{i}=<TP_{1},TP_{2},\ldots ,TP_{M_{1}},FP_{1},FP_{2},\ldots ,FP_{M_{2}}>\\ \;=<P_{1},P_{2},\ldots ,P_{M}> \end{array}$$

Based on Equation 10, we can further obtain a feature matrix for all *N* proteins in *G* as follows:


(11) $$Z={\left[\begin{array}{cc} V_{1}\cdots & V_{N} \end{array}\right]}^{T}={\left[z_{ij}\right]}_{N\times M}$$

Based on Equation 11, it is obvious that we can adopt entropy to measure the weight of each feature in all *M* different features as follows:


(12) $$w_{j}=\left(1-e_{j}\right)/\sum_{i=1}^{M}\left(1-e_{i}\right)$$

Where,


(13) $$e_{j}=-\sum_{i=1}^{N}z_{ij}\ln z_{ij}/\ln N$$

Moreover, according to Equation 13, we can further calculate the feature-based score of *p*_*i*_ for any given protein as follows:


(14) $$CScore\left(p_{i}\right)=\sum_{j=1}^{M}w_{j}Z_{ij}$$

Based on Equation 14, we can construct a new matrix *H* as follows:


(15) $$H_{ij}=\left\{ \begin{array}{cc} \frac{CScore\left(p_{i}\right)}{\sum_{l=1}^{N}CScore\left(p_{l}\right)}; & ifi=j\\ \frac{min\left\{CScore\left(p_{i}\right),CScore\left(p_{j}\right)\right\}}{\sum_{l=1}^{N}CScore\left(l\right)}; & else \end{array}\right.$$

Hence, according to Equation 15, we can obtain stable scores for all proteins in an iterative way as follows:


(16) $$Y^{t+1}=\alpha HY^{t}+\left(1-\alpha \right)Y^{0}$$

Where the parameter α ∈ (0, 1) and *Y*^0^ = < *FP*_2_(*p*_1_), *FP*_2_(*p*_2_), …, *FP*_2_(*p*_*N*_) > is the vector consisting of initial scores of all proteins. Moreover, for convenience, we define the final stable scores obtained by Equation 16 as *Y^Final^*.

#### Further Optimization

Proteins can be considered as data points in the feature space, and how to predict the similarity between potential essential proteins in the feature space is very important for the prediction of essential proteins. Wang and Zhang (2008) found that every data point in a high-dimensional space can be reconstructed by its neighbors. Zhang et al. (2017) proposed a new similarity measure to predict drug side effects based on characteristics of drugs. Hence, based on above concepts, in this section, we will first define protein nodes in the original PPI network as data points, and the gene expression data of proteins as features of corresponding data points. And for convenience, for any given protein *p*_*i*_, let *g*_*i*_ = < *g*_*i*1_, *g*_*i*2_, …, *g*_*i*36_ > represent its gene expression data, where *g*_*it*_ represents the gene expression level of *p*_*i*_ at the *t*th time point, then, we can further reconstruct each data point *p*_*i*_ based on features of its neighbors by minimizing the following reconstruction error ε_*i*_:


(17) $$\begin{array}{c} \varepsilon_{i}=||g_{i}-\sum_{p_{j}\in NG\left(p_{i}\right)}s_{i,j}g_{j}^{2}||+||s_{i}^{2}||\\ \;=||\sum_{p_{j}\in NG\left(p_{i}\right)}s_{i,j}{\left(g_{i}-g_{j}\right)}^{2}||+\sum_{p_{j}\in NG\left(p_{i}\right)}{\left(s_{i,j}\right)}^{2}\\ \;=\sum_{p_{j},p_{k}\in NG\left(p_{i}\right)}s_{i,j}s_{i,k}{\left(g_{i}-g_{j}\right)}^{T}\left(g_{i}-g_{j}\right)+\sum_{p_{j}\in NG\left(p_{i}\right)}{\left(s_{i,j}\right)}^{2}\\ \;=\sum_{p_{j},p_{k}\in NG\left(p_{i}\right)}s_{i,j}\left(G^{i}+I\right)s_{i,k}\\ \;=s_{i}^{T}\left(G^{i}+I\right)s_{i}\\ s.t.\sum_{p_{j}\in NG\left(p_{i}\right)}s_{i,j}=1,s_{i,j}\geq 0 \end{array}$$

Here, *G*^*i*^ = (*g*_*i*_−*g*_*j*_)^*T*^(*g*_*i*_−*g*_*j*_), *s*_*i*_ = (*s*_*i*,1_, *s*_*i*,2_⋯*s*_*i*, *k*_)*^T^*, $||g_{i}-\sum_{p_{j}\in NG\left(p_{i}\right)}s_{i,j}g_{j}^{2}||$ is the item of reconstruction error, $||s_{i}^{2}||$ is used for regularization and *I* is the identity matrix.

Obviously, according to Equation 17, let $S_{i,j}=\{\begin{array}{ll} s_{i,j}: & if\;p_{j}\in NG(p_{i})\\ 1: & i=j\\ 0: & otherwise \end{array}$, then we can obtain a *N* × *N*-dimensional similarity matrix *S* as follows:


(18) $$S=\left[\begin{array}{ccc} S_{11} & \cdots & S_{1N}\\ \vdots & \ddots & \vdots \\ S_{N1} & \cdots & S_{NN} \end{array}\right]$$

In addition, for any given protein node *p*_*i*_ in *G*, we can calculate the similarity *s*_*i,j*_ between it and its neighboring node *p*_*j*_ ∈ *NG*(*p*_*i*_) as follows:


$$\begin{array}{ccc} min & & s_{i}^{T}\left(G^{i}+\mu I\right)s_{i}\\ s.t. & & \sum_{p_{j}\in NG\left(p_{i}\right)}s_{i,j}=1,s_{i,j}\geq 0 \end{array}$$

Thereafter, let *T*^0^ = *Y^Final^*, based on above newly obtained matrix *S*, we can further optimize the scores for all proteins in an iterative way as follows:


(19) $$T^{\sigma +1}=\beta ST^{\sigma }+\left(1-\beta \right)T^{0}$$

Here, there is β ∈ (0, 1).

Based on the above descriptions, the process of LNSPF can be described in detail as follows:

**Algorithm:** LNSPF.

**Input:** Original PPI network, gene expression data, subcellular location data and homologous data, parameters δ and *K.*

**Output:** Rank the proteins in descending order according to *T^Final^* value, and output TOP K%.

**Step 1:** According to Equations 3, 4, an original PPI network *G* = (*V*, *E*) is generated, based on which, topological features are extracted;

**Step 2:** According to Equations 7, 9, functional characteristics are extracted from the subcellular location data and homologous data, respectively.

**Step 3:** According to Equation 15, the matrix *H* is obtained;

**Step 4:** let *t* = *t* + 1; calculate *Y*^*t* + 1^ according to Equation 16;

**Step 5:** Repeat step 4 until ||*Y*^*t* + 1^−*Y*^*t*^|| < δ, the matrix *Y^Final^* is obtained;

**Step 6:** According to Equation 18, the similarity matrix *S* is obtained;

**Step 7:** let *T*^0^ = *Y^Final^* and σ = σ + 1, the matrix *Y^Final^* is further optimized according to Equation 19;

**Step 8:** Repeat step 7 until ||*T*^σ + 1^−*T*^σ^|| < δ, the matrix *T^Final^* is obtained;

**Step 9:** The values of *T^Final^* are sorted in descending order, and the top K% proteins with the highest final scores are output.

## Experimental Results

### Experimental Data

During experiments, we first downloaded known PPIs from three different databases such as the Gavin (Gavin et al., 2006) database, the DIP (Xenarios et al., 2002) database, and the Krogan (Cherry, 1998) database, and then, after filtering repeated interactions and self-interactions, we finally obtained 24,743 interactions between 5,093 proteins based on the DIP database, 7,669 interactions between 1,855 proteins based on the Gavin database, and 14,317 interactions between 3,672 proteins based on the Krogan database, respectively. Moreover, we obtained a group of 1,285 essential proteins in Saccharomyces cerevisiae from the databases of SGDP (Holman et al., 2009), SGD (Holman et al., 2009), DEG (Zhang and Lin, 2009), and MIPS (Bruno et al., 2012) as well. Furthermore, we downloaded the homology information of proteins from the Inparanoid database (Gabriel et al., 2010), the gene expression dataset composing of 6,776 proteins representing the gene expression level of proteins in continuous metabolic cycles from the database provided by Tu et al. (2005), and the dataset of subcellular location information from the part-means database (Binder et al., 2014) separately. Especially, the dataset of subcellular location information consists of 11 kinds of subcellular localization, namely, the extracellular, peroxisome, nucleus, plasma, endosome, mitochondrion, vacuole, cytosol, golgi, cytoskeleton, and endoplasmic, which are closely related to known key proteins. At last, to evaluate the recognition rate of true essential proteins predicted by LNSPF, we compared LNSPF with 16 representative predictive models, as shown in Table 1, namely, DC, EC, CC, IC, SC, BC, NC, Pec, LAC, CoEWC, POEM, ION, TEGS, RWHN, IoMCD, and CVIM.

**TABLE 1:** A brief description of the existing representative prediction models.

Algorithm	Network topology	Biological information	Particular year
DC (Hahn and Kern, 2005)	Degree centrality	NO	2005
EC (Bonacich, 1987)	Eigenvector centrality	NO	1987
CC (Wuchty and Stadler, 2003)	Closeness centrality	NO	2003
IC (Stephenson and Zelen, 1989)	Information centrality	NO	1989
SC (Estrada and Rodríguez-Velázquez, 2005)	Subgraph centrality	NO	2005
BC (Joy et al., 2005)	Betweenness centrality	NO	2005
NC (Wang et al., 2012)	Neighbor centrality	NO	2012
PEC (Li et al., 2012)	Edge clustering coefficient	Gene expression data	2012
LAC (Li et al., 2015)	Degree centrality, common neighbor node	NO	2011
CoEWC (Zhang et al., 2013)	Clustering coefficient	Gene expression data	2013
POEM (Zhao et al., 2014)	Degree centrality, subgraph, edge clustering coefficient, closeness centrality	Gene expression data	2014
ION (Peng et al., 2015a)	Edge clustering coefficient	Orthologous data	2012
TEGS (Li et al., 2018)	Subnetwork partition and prioritization	subcellular localization data	2018
RWHN (Zhao et al., 2019)	Degree centrality, protein-domain	Orthologous data, subcellular localization	2019
IoMCD (Sun et al., 2021)	Common neighbor node, degree Centrality	Gene expression data, orthologous data	2021
CVIM (Li et al., 2020)	Degree centrality, common neighbor node	Gene expression data, orthologous data	2020

### Influence of Parameters on Linear Neighborhood Similarity-Based Protein Multifeatures Fusion Performance

In LNSPF, we set parameters α and β, the value ranges of both α and β are (0, 1), to adjust the final protein score. During experiments, we will set different values to the parameter α or β first based on the Gavin database and the DIP database, respectively, and then, the setting value with the highest prediction accuracy of essential protein will be selected as the final value of parameter α or β. Based on the Gavin dataset, we set α to 0.1., 0.8, and 0.9 to predict the effect of the preliminary iterative algorithm. From observing Table 2, it is obvious that when α = 0.6, the protein score with obvious effect and the most stable one can be obtained. At this time, the setting value of α in Gavin dataset is 0.6 and that in DIP database is 0.8. β set 0.1, …, 0.8, 0.9. The prediction results based on Gavin data set (α = 0.6) and dip data set (α = 0.8) are shown in Tables 3, 4, respectively. By observing Table 3, it is easy to see that the prediction performance of LNSPF is the highest at 1%, 5%, 15%, 20%, and 25% when β = 0.4 is used. Therefore, based on Gavin data set, it is appropriate to set β as 0.4. By observing Table 4, it is easy to see that the prediction performance of LNSPF is the highest at 1%, 5%, 10%, and 25% when β = 0.2 is used. Therefore, based on the DIP data set, it is more appropriate to set β as 0.2.

**TABLE 2:** Influence of parameter α on the effect of initial iteration algorithm in Gavin database.

	α								
Rank	0.1	0.2	0.3	0.4	0.5	0.6	0.7	0.8	0.9
Top1% (19)	16	17	18	18	18	**18**	17	15	15
Top5% (93)	75	80	83	83	82	80	80	78	79
Top10% (186)	147	155	156	159	162	162	**163**	160	161
Top15% (278)	198	205	213	219	218	**220**	220	219	217
Top20% (371)	249	259	264	268	271	267	**274**	278	272
Top25% (464)	303	306	309	314	317	**322**	322	320	321

*The bold values represent the best predictive performance achieved by LNSPF under different conditions.*

**TABLE 3:** Effect of parameter β on prediction performance of LNSPF in Gavin database.

	β								
Rank	0.1	0.2	0.3	0.4	0.5	0.6	0.7	0.8	0.9
Top1% (19)	18	**19**	**19**	**19**	18	18	18	18	17
Top5% (93)	81	83	83	**84**	82	82	82	81	78
Top10% (186)	164	**165**	164	163	164	166	164	163	161
Top15% (278)	221	**223**	221	**223**	222	219	220	219	210
Top20% (371)	271	274	274	**278**	274	272	272	270	262
Top25% (464)	324	324	325	**326**	325	321	319	314	310

*The bold values represent the best predictive performance achieved by LNSPF under different conditions.*

**TABLE 4:** Effect of parameter β on prediction performance of LNSPF based on DIP database.

	β								
Rank	0.1	0.2	0.3	0.4	0.5	0.6	0.7	0.8	0.9
Top1% (51)	46	**47**	47	46	46	46	44	44	43
Top5% (255)	203	**208**	205	203	203	200	198	197	189
Top10% (510)	347	**352**	350	**352**	**352**	349	342	334	330
Top15% (764)	468	468	467	**469**	467	459	457	458	429
Top20% (1019)	547	546	544	**548**	547	542	542	535	519
Top25% (1274)	626	**630**	628	625	622	622	623	615	608

*The bold values represent the best predictive performance achieved by LNSPF under different conditions.*

## Comparison of LNSPF With Other Methods

### Comparison of the Number of Real Essential Proteins Between Linear Neighborhood Similarity-Based Protein Multifeatures Fusion and 14 Representative Methods

**FIGURE 2:**
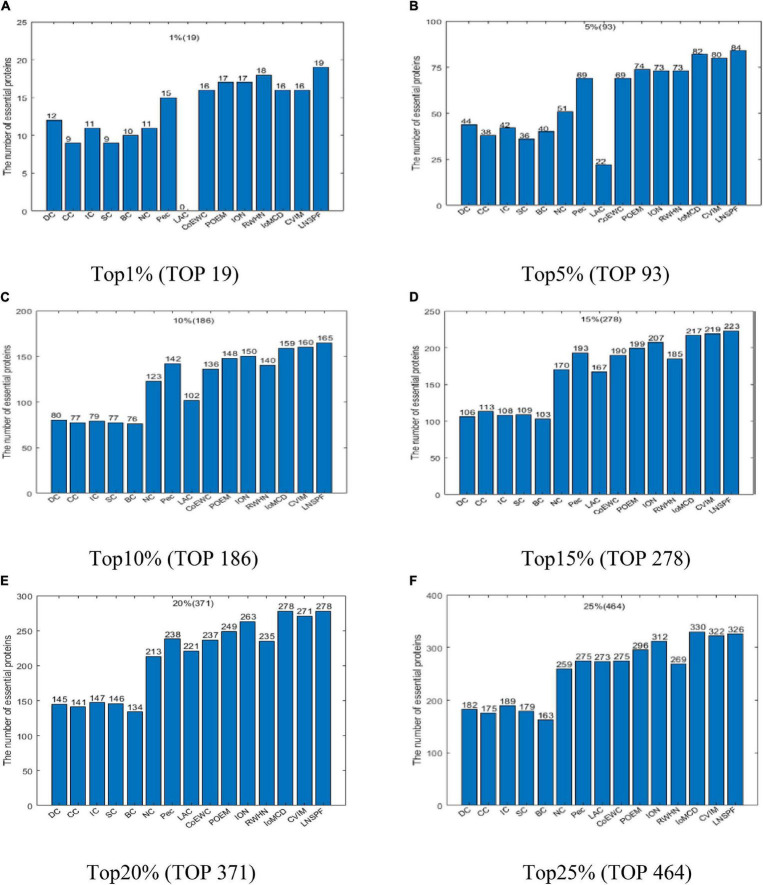
Comparison results of the numbers of real key proteins predicted by LNSPF, DC, CC, IC, SC, BC, NC, PEC, LAC, CoEWC, POEM, ION, RWHN, IoMCD, and CVIM based on the GAVIN database. **(A)** Top 1% ranked proteins. **(B)** Top 5% ranked proteins. **(C)** Top 10% ranked proteins. **(D)** Top 15% ranked proteins. **(E)** Top 20% ranked proteins. **(F)** Top 25% ranked proteins.

According to above descriptions, it is easy to see that LNSPF can achieve it best predictive performance while we set α to 0.6 and β to 0.4 based on the Gavin database. Hence, in this section, in order to estimate the actual predictive performance of LNSPF, we will first compare it with 14 advanced predictive methods based on the Gavin database while setting α to 0.6 and β to 0.4, and the comparison results are shown in Figure 2. From observing the Figure 2, it is easy to see that, in the ranking of the number of true essential proteins inferred by these 15 predictive methods, LNSPF can achieve better predictive performance than all these competitive methods in top 1, 5, 10, 15, and 20% predicted key proteins simultaneously. For instance, from the top 1% to top 20% predicted key proteins, the predictive accuracies of LNSPF are 15.8, 4.3, 2.6, 1.4, and 1.8% higher than that of the method of CVIM, respectively.

**FIGURE 3:**
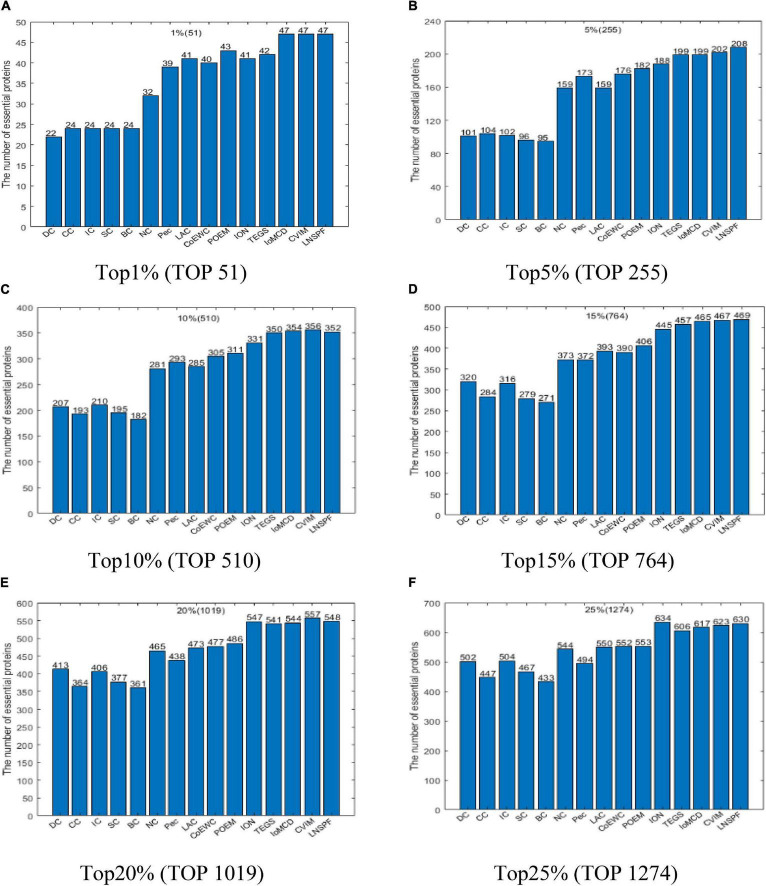
Comparison results of the numbers of real key proteins predicted by LNSPF, DC, CC, IC, SC, BC, NC, PEC, LAC, CoEWC, POEM, ION, RWHN, IoMCD, and CVIM based on the DIP database. **(A)** Top 1% ranked proteins. **(B)** Top 5% ranked proteins. **(C)** Top 10% ranked proteins. **(D)** Top 15% ranked proteins. **(E)** Top 20% ranked proteins. **(F)** Top 25% ranked proteins.

Similarly, according to above descriptions, it is easy to see that LNSPF can achieve it best predictive performance while we set α to 0.6 and β to 0.2 based on the DIP database. Hence, in this section, in order to estimate the actual predictive performance of LNSPF, we will further compare it with 14 advanced predictive methods based on the DIP database while setting α to 0.6 and β to 0.2, and the comparison results are shown in Figure 3. From observing the Figure 3, it is easy to see that, the numbers of essential proteins detected by LNSPF in the top 1, 5, 10, 15, 20, and 25% ranked proteins are significantly better than that of all competitive methods as a whole.

### Receiver Operating Characteristic Curve Verification

**FIGURE 4:**
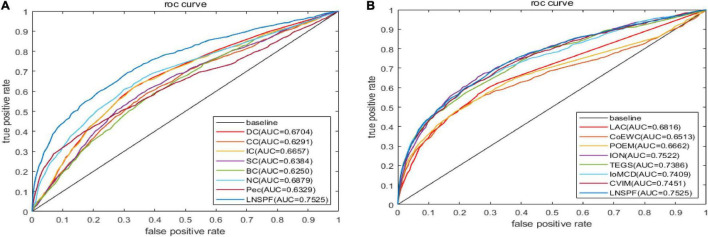
The ROC curves of LNSPF method based on DIP dataset and DC, CC, IC, SC, BC, NC, Pec, and LAC CoEWC, POEM, ION, TEGS, IoMCD, and CVIM 14 prediction methods. **(A)** Comparison between LNSPF and DC, CC, IC, SC, BC, NC, PEC. **(B)** Comparison between LNSPF and LAC, CoEWC, POEM, ION, TEGS, IoMCD, CVIM.

**FIGURE 5:**
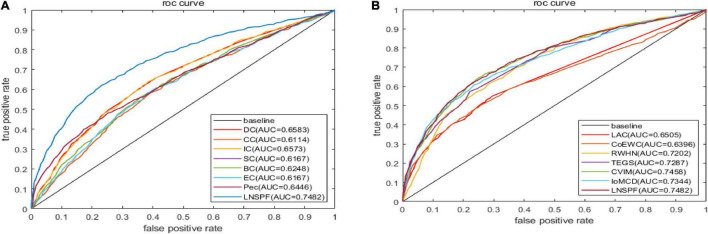
The ROC curves of LNSPF method based on Krogan dataset and DC, CC, IC, SC, BC, EC, PEC, and LAC, CoEWC, RWHN, TEGS, CVIM, and IOMCD 13 prediction methods. **(A)** Comparison between LNSPF and DC, CC, IC, SC, BC, EC, PEC. **(B)** Comparison between LNSPF and LAC, CoEWC, RWHN, TEGS, CVIM, IOMCD.

Receiver operating characteristic curve (ROC) is used to compare the prediction performance of LNSPF with DC, CC, IC, SC, BC, NC, PEC, LAC, CoEWC, POEM, ION, TEGS, IoMCD, and CVIM based on DIP data set. The larger the area of ROC curve, the better the performance of the model, it can be seen from Figure 4 and Table 5 that the performance of this model is significantly higher than that of the 14 competitive methods. The prediction performance of LNSPF method based on Krogan dataset compared with DC, CC, IC, SC, BC, EC, PEC, and LAC, CoEWC, RWHN, TEGS, CVIM, and IoMCD 13 competing methods. It can be seen from Figure 5 and Table 6 that the performance of this model is significantly higher than that of these 13 competing methods.

**TABLE 5:** Based on DIP database, LNSPF and AUC of 14 competitive methods.

Method	LNSPF	DC	CC	IC	SC	BC	NC	Pec
AUC	0.7525	0.6704	0.6293	0.6657	0.6384	0.6250	0.6879	0.6329

**Method**	**LNSPF**	**LAC**	**CoEWC**	**POEM**	**ION**	**TEGS**	**IoMCD**	**CVIM**

AUC	0.7525	0.6816	0.6513	0.6662	0.7522	0.7386	0.7409	0.7451

**TABLE 6:** AUC values of LNSPF and 13 competing methods based on Krogan dataset.

Method	LNSPF	DC	CC	IC	SC	BC	EC
AUC	0.7482	0.6583	0.6114	0.6573	0.6167	0.6248	0.6167

**Method**	**PEC**	**LAC**	**CoEWC**	**RWHN**	**TEGS**	**CVIM**	**IoMCD**

AUC	0.6446	0.6505	0.6396	0.7202	0.7287	0.7458	0.7344

### Verification of Jackknife Method

**FIGURE 6:**
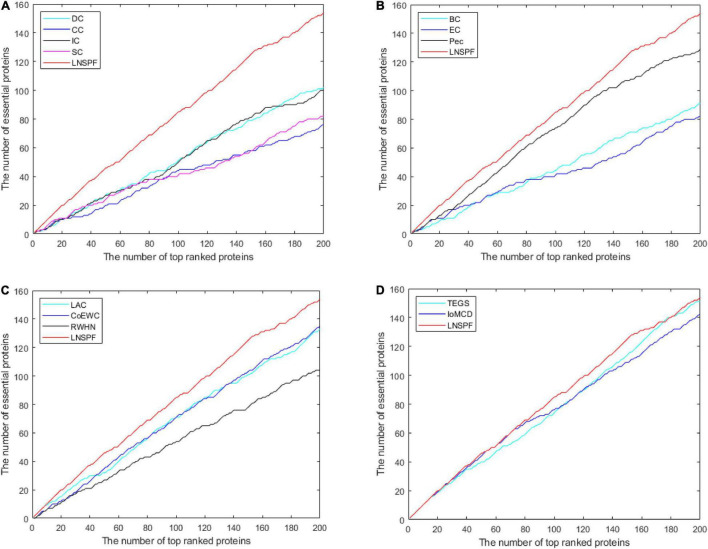
The figure shows the Jackknife curves of LNSPF and DC, CC, IC, SC, BC, EC, and PEC based on Krogan dataset, and LAC, CoEWC, RWHN, TEGS, and IOMCD 12 prediction methods. The X-axis represents the number of potentially critical proteins ranked in the top 200, and the Y-axis represents the number of truly essential proteins identified by these models. **(A)** Comparison between LNSPF and DC, CC, IC, SC. **(B)** Comparison between LNSPF and BC, EC, PEC. **(C)** Comparison between LNSPF and LAC, CoEWC, RWHN. **(D)** Comparison between LNSPF and TEGS, IOMCD.

**FIGURE 7:**
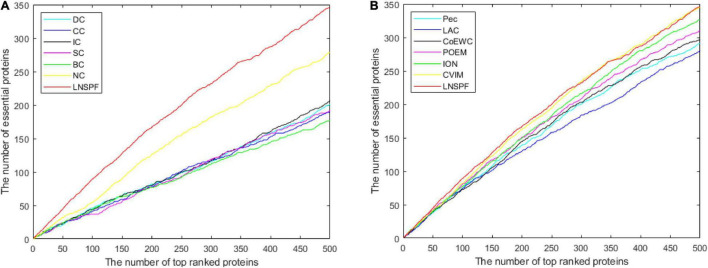
The figure, respectively, shows the Jackknife curve of LNSPF and DC, CC, IC, SC, BC, NC, and PEC, LAC, COEWC, POEM, ION, and CVIM 12 prediction methods based on DIP data set. The X-axis represents the number of potentially critical proteins ranked in the top 500, and the Y-axis represents the number of truly essential proteins identified by these models. **(A)** Comparison between LNSPF and DC, CC, IC, SC, BC, NC. **(B)** Comparison between LNSPF and PEC, LAC, COEWC, POEM, ION, CVIM.

In this section, I’ll use the Jackknife method to verify the performance of the LNSPF against the other models. The performance of LNSPF was compared with DC, CC, IC, SC, BC, EC, PEC, and LAC, CoEWC, RWHN, TEGS, and IOMCD based on Krogan data set. As shown in Figure 6. It is obvious that this method is superior to other models. The performance of LNSPF is compared with DC, CC, IC, SC, BC, NC, PEC, and LAC, COEWC, POEM, ION, and CVIM based on DIP data set, as shown in Figure 7.

## Discussion

Essential proteins play an important role in cell growth and regulation, for the past few years, accumulating computational methods have been proposed to detect potential key proteins, however, the predictive performances of these existing methods are not very satisfactory yet. In this study, a novel predictive model called LNSPF was designed by combining topological features of PPI networks with a series of biological characteristics of proteins to detect potential key proteins. In LNSPF, a new entropy-based method for feature fusion and a linear neighborhood similarity method for optimization were adopted. Comparing with traditional identification methods, LNSPF can achieve better predictive performance, which demonstrates that the method based on the fusion of biological information of proteins and topological features of PPI networks can improve the prediction accuracy of essential proteins effectively. In addition, there are some limitations in current version of LNSPF as well, for example, the loss of gene time expression data or homologous data of some proteins will affect the recognition accuracy of LNSPF to some degree.

## Conclusion

In this paper, an iterative model of protein multifeature fusion based on linear neighborhood similarity (LNSPF) is proposed to predict essential proteins by fusing biological and topological information of proteins. In LNSPF, first, the topological features are extracted from the original PPI network, and then the functional features are extracted from the subcellular location data. Second, an entropy weight method is used to fuse the features, and then a stable protein score is obtained by an iterative method. At last, a linear neighborhood similarity method is used to optimize the score effectively. The experimental results show that based on Gavin data sets, the Krogan data sets, and DIP held several experimental data sets, through a variety of methods to verify the effectiveness of the new model LNSPF and stability. Compared with many advanced prediction models, the new model LNSPF has better prediction effect.

## Data Availability Statement

The original contributions presented in the study are included in the article/supplementary material, further inquiries can be directed to the corresponding author/s.

## Author Contributions

XZ and YZ conceived the study, implemented the algorithms corresponding to the study, and wrote the manuscript. LW and ZC improved the study based on the original model. YT and LW supervised the study. XZ and YZ revised the manuscript. All authors reviewed and improved the manuscript.

## Conflict of Interest

The authors declare that the research was conducted in the absence of any commercial or financial relationships that could be construed as a potential conflict of interest.

## Publisher’s Note

All claims expressed in this article are solely those of the authors and do not necessarily represent those of their affiliated organizations, or those of the publisher, the editors and the reviewers. Any product that may be evaluated in this article, or claim that may be made by its manufacturer, is not guaranteed or endorsed by the publisher.
